# Changes in the communicative skills of young people as a result of a communication training

**DOI:** 10.1590/2317-1782/20232022041

**Published:** 2023-09-01

**Authors:** Maria Lucia Graziano Magalhães Torres, André Luiz Lopes Sampaio, Hugo Cesar Pinto Marques Caracas

**Affiliations:** 1 Laboratório de Ensino e Pesquisa em Otorrinolaringologia, Universidade de Brasília - Brasília (DF), Brasil.; 2 Instituto de Gestão Estratégica em Saúde, Hospital de Base do Distrito Federal - Brasília (DF), Brasil.

**Keywords:** Communication, Communication Course, Teenagers, Training, Skills, Comunicação, Curso de Comunicação, Adolescentes, Treinamento, Habilidades

## Abstract

**Purpose:**

To analyze the effect of communication training developed for adolescents on the youth’s communication skills.

**Methods:**

Forty-one young people participated in the study and were divided into two groups. Group I received intervention - communication training for five weeks; Group II - were guided on the importance of voice for communication. Participants had their communication skills assessed before/after interventions through a self-administered questionnaire, answered by the participant and parents. Both groups had oral presentations recorded on video, which were analyzed by speech therapists through the evaluation of the communication profile, listing the aspects that should be observed as a point of strength/opportunity for improvement. Association between qualitative variables was assessed using Fischer’s exact test, Pearson’s chi-square test, McNemar’s test, and McNemar-Bowker’s test. To compare groups, paired t-test and t-test for independent samples, p level <0.05, were used.

**Results:**

The analysis of questionnaires showed improvement in GI in two of the four skills assessed and no difference for GII. The video evaluations showed a significant difference between the groups in terms of eye contact and speech velocity.

**Conclusion:**

This study points out that the communication training method applied to young people might improve communicative skills, providing some insights into their communication strengths and potential areas for development.

## RESUMO

**Objetivo:**

Analisar o efeito de um treinamento em comunicação desenvolvido para adolescentes nas habilidades comunicativas dos jovens.

**Método:**

Quarenta e um jovens participaram do estudo divididos em dois grupos. Grupo I – treinamento de comunicação por duas semanas; Grupo II – palestra sobre importância da voz. Os participantes tiveram suas habilidades comunicativas avaliadas antes e após as intervenções mediante questionário autoaplicado respondido pelos jovens e seus pais. Ambos os grupos tiveram apresentações orais gravadas antes e após as intervenções, que foram avaliadas por fonoaudiólogos através de questionário, listando características da comunicação. A associação entre variáveis qualitativas foi verificada usando teste exato de Fisher, qui-quadrado de Pearson, teste de McNemar e teste de McNemar-Bowker. Para comparação entre os grupos, foram usados teste t para amostras pareadas e teste t para amostras independentes com nível de significância de 95%.

**Resultados:**

A análise dos questionários mostrou melhora do Grupo I em duas das quatro habilidades avaliadas, não havendo diferença no Grupo II. A avalição dos vídeos mostrou diferença significativa entre os grupos no contato visual e na velocidade da fala.

**Conclusão:**

O estudo mostrou que o método de treinamento de comunicação aplicado aos jovens, melhorou habilidades comunicativas, fornecendo autoconhecimento sobre seus pontos fortes e pontos que podem ser melhorados.

## INTRODUCTION

A communication skill is the decoding of the meaning of sent and received messages, establishing a plan of thought that is adequate and consistent with the communicator’s needs. It is a process of understanding and sharing messages sent and received that influence the individuals’ behavior, contributing to people being constantly involved in an interactional field^(1)^.

Communication skills are one of the most important soft skills and are observed and developed from the visual, auditory, and kinesthetic communication channels. People who develop such skills are known as competent communicators. They are usually described as someone able to choose and adequately perform a wide range of behaviors, to see the perspectives of other individuals, besides being committed to important relationships^(2)^. Consequently, good oral communication skills will improve social skills such as giving and receiving feedback^(3)^.

On the other hand, the lack of communication skills reduces the expressiveness of public speech^(4)^. When speaking in public, shy people usually have somatic symptoms, such as facial flushing, tremors, palpitations, and dry mouth^(5)^. They also present altered nonverbal aspects of communication, such as a lack of voice projection, reduced voice volume, accelerated speech rate, lack of eye contact with the interlocutor, restrained behavior, withdrawn gestures, and tense posture^(4,6,7)^. Another study concluded that shy students participate less in activities in public, are less likely to volunteer, and feel more inhibited than students who are not shy^(8)^.

In the authors’ experience over 35 years, we observed that a significant number of adult clients identified themselves as shy. They reported their difficulties in speaking in public and communicating with their peers since adolescence and have the perspective that those difficulties impaired their performance at their current work. When they did not refer to themselves, they always talked about someone close, such as brothers or best friends at school. Systematically, people sighed: “Oh, if only I had had the opportunity to learn about personal and communication skills when I was a teenager!”

Communication skills are not just a personal trait; they are a series of modifiable skills that can be developed so that the individual becomes a better communicator^(9)^. So, individuals with communication difficulties can communicate better after participation in communication training courses^(10-12)^. This procedure is used to ensure better interaction between people. Practical and theoretical training can lead to meaningful learning^(13)^.

A training course is a known and reliable method for improving communication skills between individuals in many aspects of their lives. For example, training for nurses in communication led to the acquisition of skills to establish better and more effective communication with the patient and other members of the medical team during nursing education. So, they can experience these later in practice in a clinical setting^(13)^.

Several other communication skill training initiatives with positive results have been recorded in the literature^(14,15)^. However, they are available only for adults. Additionally, except for the training of doctors and nurses previously mentioned^(3,11,13,14)^, no other studies were found to present the results of these training initiatives. It is necessary to build a bridge of knowledge with references about young people and their communication difficulties, considering the psychological aspects, which are quite peculiar to this age group. No detailed description of a method for training youth communication was found in the literature. Therefore, there is a need to develop and analyze the effects of a proposal for training youth communication because, at that age, the possibilities for learning are entirely open.

The method was created to provide young people with a good understanding of their communication difficulties and how to overcome them.

It is known that shyness is considered a common trait of the human personality, manifested by cognitive, somatic, and behavioral symptoms^(6,16)^, and is not considered a pathology^(5,6,16)^. Studies indicate that shyness is prevalent in young university students who participate in only a few public speaking activities, are afraid to speak in public, report to speak with low intensity, and cannot use their hands naturally during public presentations^(17)^.

Nonverbal aspects in communication such as voice, speech rate, gestures, or facial expressions directly influence speech^(18)^ and give meaning to what is said^(18,19)^.

Therefore, the objective of this work is to analyze the effect of the training on communication skills in young people.

## METHODS

This study was carried out according to the World Medical Association’s Declaration of Helsinki for experiments involving humans. It was approved by the Human Research Ethics Committee of the Instituto de Gestão Estratégica do Distrito Federal (CAAE: 38435020.9.0000.8153; opinion 4.355.357). All participants and their guardians read and signed the consent and/or assent form.

All participants were recruited from a private school in Planaltina - DF - Brazil, through an invitation made by the school’s coordination to the groups of mothers, fathers, or guardians of young people in the age group included in the study.

The inclusion criteria were the age group of 13 to 21 years old of both genders. Individuals diagnosed with communication disorders or with evident disorders in communication such as stuttering, autism, Asperger syndrome, mental disability, and who have impaired communication due to a clinical condition were excluded from the research. However, no participant met the exclusion criteria.

The sample was initially composed of 60 young people of school age with an average age between 13 and 17 years old. They were allocated through simple randomization into two groups: Group I (GI) - 30 young people who received the communication training intervention; and Group II (GII) - 30 young people who watched lectures on the importance of the voice in communication. Interventions in both groups were performed by the same speech therapist, always after their regular activities. The interventions carried out in GI and GII are discussed, respectively.

Nineteen students did not complete all the stages of the study, and they were excluded. As a result, GI had a total of 20 subjects, and GII had 21 subjects. The allocation randomization was carried out by a draw from an opaque white envelope, which contained 30 pieces of paper with “I” written on them and 30 written papers with “II.” The researcher carried out the draw after the initial evaluation according to the inclusion criteria. So, the number designated the group in which the subject would participate. The name of the participant, the allocation group, and the identification code were recorded in an Excel® spreadsheet by one assistant.

Training, lectures, and data collection were carried out on the school premises. Before beginning the research, a meeting was held with all parents to present the objective of the research and the expected results. For the application of the selection criteria, an interview was held with the parents or guardians. At that moment, the questionnaire, the terms of consent, terms of assent, and image rights use authorization were presented.

Communication Skills Training - GI -The method used consisted of training in social presentation activities, public speaking, improvised speech, perception of the importance of the voice, expressiveness, and communicative attitudes. The training also proposes practical activities and exercises in pairs and groups. Communication Skills Training lasted 16 hours over five weeks, with five classes of three hours each. Before the training, a meeting was held with the parents to present the project and guidelines. The description of the training plan sessions regarding the objectives/theme of each meeting can be seen in Table 1.

**Table 1:** Theme/objective of each meeting during the training

**Class**	**Theme**	**Action**
**1**	• Sensitize regarding the importance of communication.	• Initial conversation
	• Self-knowledge	
	• Voice and diction	
	• Initial conversation	
**2**	• Breaking the ice	• Organizing the Speech
	• First contact	
	• Organizing the speech	
**3**	• Deepening a conversation	• Starting and finishing a conversation
	• Starting and finishing a conversation	
**4**	• Improving self-esteem	• Group conversation (meetings, friends conversation)
	• Feeling, thinking, action	
	• Posture issues	
	• Group conversation (meetings, friend’s conversation)	
	• Make a presentation or a lecture	
**5**	• New ways of communicating	• Make a presentation or a lecture
	• Final recording and self-evaluation	

Guidance on the importance of the voice in communication - GII- This intervention is a lecture on the importance of the voice in communication and tongue vibration exercises and was used as a “placebo.” During the five-week training period, this group continued to perform their usual daily activities. The participants in this GII received communication training after data collection.

The outcomes were analyzed by a self-assessment questionnaire applied to the participants. A similar questionnaire was applied to the parents to evaluate their perception of their children’s communication skills. An oral presentation recorded on video before and after the intervention was used to evaluate the communication profile of the participants.

The questionnaire applied was a version translated and cross-culturally adapted to the Portuguese language of the “Interpersonal Communication Skills Inventory”^(20)^. It is a self-administered questionnaire that has 40 items called “Interpersonal Communication Skills Inventory”^(20)^. In addition to the translation into Portuguese, the back translation was carried out by a native speaker of the English language and then translated back into Portuguese. The response categories of the instruments are: “frequently,” “sometimes,” and “rarely.” The answers were scored on a Likert scale from 0 to 3. The items are divided into four sections, with ten questions each, which assess the following skills: I - the ability to send a clear message; II - the ability to listen; III - the ability to give and receive feedback; IV - the ability to deal with emotions. The guardians of the young people from both GI and GII answered the parental assessment questionnaires at the same time as the participants’ self-assessment, before and after the training.

The recorded oral presentation consisted of the reproduction of a short story of one-minute duration, which was previously read. The story of the first presentation was different from the story of the second presentation. The oral presentation was recorded on video by a portable Sony® camcorder. The videos were arranged in pairs and randomized as to the moment of evaluation and the type of intervention to blind the evaluators’ judgment. To evaluate the videos, three speech therapists specialized in voice were selected. They did the evaluation by filling in an objective form about the aspects of communication: voice quality, diction, body and facial expressiveness, communicative attitudes, feet tension, and body tension. The videos were sent randomly in a USB encrypted pen drive and numbered by pairs A and B, from 1 to 50.

The data were analyzed in a descriptive and inferential manner. The SPSS 25.0 software was used. The description of the qualitative nominal variables was carried out by relative frequency and percentage frequency. The quantitative variables were described using measures of variability (standard deviation), central tendency (mean and median), and position (minimum, maximum, first quartile, and third quartile).

The inferential analysis of the association between the qualitative nominal variables of two categories and the independent groups was performed with Fisher’s Exact Test and that of the multiple categories and independent groups, with Pearson’s chi-squared test. The association between the qualitative nominal variables of two categories and the dependent groups was carried out with the McNemar’s test, and the multiple categories and dependent groups were performed using the McNemar-Bowker test. The associations were tested for the variables of body posture, feet position, diction, voice, intonation, speed, pauses, head movement and look before and after the intervention.

The quantitative variables went through an analysis of the homogeneity of the distribution using the Shapiro Wilk test, and all of them obtained normal distribution. The comparison between the two intervention groups was performed with the Independent T-Test, and the comparison between the evaluation moments was performed with the paired t-test. A significance level of 5% was considered in all inferential analyses. The sum was performed for each of the skills assessed through the self-assessment questionnaire of communication skills to perform the intergroup and intragroup comparison. The homogeneity of the groups was evaluated before and after the intervention. The answers of the participants’ and parents’ self-assessment questionnaires were compared within each group before and after the intervention.

## RESULTS

The mean age and the standard deviation for GI and GII were 14.55 (1.19) and 14.67 (1.28), respectively. The gender distribution between the groups was nine females in both groups and eleven males for GI and twelve males for GII. In comparing the groups regarding age and sex, there was no difference in either characteristic (p=0.764 and p=1.0, respectively).

Regarding the evaluation carried out through the questionnaire, there was no difference between the groups in any of the sections before the intervention. After the intervention, there was a difference between the groups in Section I, with higher results in GI (Table 2).

**Table 2:** Analysis of the communication skills assessment questionnaire according to the intervention group in young people

Variable	Group	Mean	SD	p-value
Score	GI	12.95	4.62	0.368
Section I (before)	GII	14.24	4.44	
Score	GI	18.90	3.26	0.030*
Section I (after)	GII	16.05	4.74	
Score	GI	15.85	4.76	0.660
Section II (before)	GII	15.24	4.02	
Score	GI	17.85	4.66	0.251
Section II (after)	GII	16.10	4.98	
Score	GI	15.35	4.78	0.677
Section III (before)	GII	14.76	4.16	
Score	GI	17.75	4.15	0.202
Section III (after)	GII	15.90	4.94	
Score	GI	13.90	6.05	0.302
Section IV (before)	GII	12.14	4.53	
Score	GI	15.45	6.57	0.115
Section IV (after)	GII	12.48	5.10	
PARENTS Score	GI	11.50	4.05	0.076
Section I (before)	GII	13.81	4.07	
PARENTS Score	GI	15.10	5.10	0.951
Section I (after)	GII	15.19	4.29	
PARENTS Score	GI	14.75	4.61	0.512
Section II (before)	GII	15.81	562	
PARENTS Score	GI	16.55	3.71	0.360
Section II (after)	GII	15.33	4.66	
PARENTS Score	GI	12.65	4.44	0.172
Section III (before)	GII	14.38	3.43	
PARENTS Score	GI	14.30	3.70	0.546
Section III (after)	GII	13.52	4.43	
PARENTS Score	GI	11.05	4.71	0.217
Section IV (before)	GII	13.24	6.36	
PARENTS Score	GI	12.45	5.00	0.966
Section IV (after)	GII	12.38	5.45	

Independent samples T-Test

* p<0.05

**Caption:** GI = Group I; GII = Group II; SD = Standard Deviation

When the answers to the questionnaires were compared before and after the training, there was an increase in the scores of sections I (p = 0.001) and III (p = 0.010) in the self-assessment of communication skills performed by young people and in sections I (p = 0.001) and III (p = 0.048) in the assessment of communication skills carried out by the parents of the young people in the GI (Table 3). The answers to both questionnaires for assessing communication skills in GII youth showed no difference before and after the lecture on the importance of the voice (Table 4).

**Table 3:** Analysis of the communication skills assessment questionnaire according to the assessment moment for Group I youth

Variable	Moment	Mean	SD	Minimum	Maximum	1Q	Median	3Q	p-value
Score	Before	12.95	4.62	7.00	24.00	8.5	13.0	16.75	0.001*
Section I	After	18.90	3.26	14.00	26.00	16.00	19.00	20.75	
Score	Before	15.85	4.76	3.00	26.00	14.00	16.00	18.75	0.074
Section II	After	17.85	4.66	12.00	29.00	13.25	17.00	22.00	
Score	Before	15.35	4,78	6.00	22.00	12.50	16.00	19.00	0.010*
Section III	After	17.75	4.15	10.00	26.00	14.25	19.,00	21.00	
Score	Before	1390	6.05	3.00	22.00	10.00	14.50	19.75	0.174
Section IV	After	15.45	6.57	3.00	24.00	11.50	16.00	21.75	
PARENTS Score	Before	11.50	4.05	6.00	21.00	8.00	11.50	14.00	0.001*
Section I	After	15.10	5.10	7.00	23.00	11.00	14.50	20.75	
PARENTS Score	Before	14.75	4.61	6.00	24.00	11.00	15.50	17.75	0.181
Section II	After	16.55	3.71	10.00	23.00	14.00	16.50	19.75	
PARENTS Score	Before	12.65	4.44	4.00	22.00	11.00	12.50	16.25	0.048*
Section III	After	14.30	3.70	8.00	19.00	10.25	15.50	17.75	
PARENTS Score	Before	11.05	4.71	2.00	22.00	8.00	10.50	14.50	0.293
Section IV	After	12.45	5.00	5.00	23.00	8.25	12.00	16.00	

Paired T-Test

* p<0.05

**Caption:** SD = Standard Deviation; 1Q = First Quartile; 3Q = Third Quartile

**Table 4:** Analysis of the communication skills assessment questionnaire according to the moment of assessment in GII youth

Variable	Group	Mean	SD	Minimum	Maximum	1Q	Median	3Q	p-value
Score	Before	14.24	4.44	6.00	22.00	10.00	14.00	19.00	0.106
Section I	After	16.05	4.74	6.00	26.00	13.00	16.00	19.50	
Score	Before	15.24	4.2	8.00	24.00	12.00	16.00	17.50	0.396
Section II	After	16.10	4.98	6.00	26.00	12.00	16.00	18.50	
Score	Before	14.76	4.16	8.00	23.00	12.00	14.00	19.50	0.164
Section III	After	15.90	4.94	8.00	24.00	12.00	16.00	20.50	
Score	Before	12.14	4.53	6.00	22.00	9.00	12.00	14.00	0.741
Section IV	After	12.48	5.10	4.00	26.00	9.50	11.00	17.00	
PARENTS Score	Before	13.81	4.07	8.00	23.00	10.00	14.00	16.50	0.270
Section I	After	15.19	4.29	9.00	22.00	12.00	14.00	20.00	
PARENTS Score	Before	15.81	5.62	5.00	26.00	11.00	17.00	20.50	0.585
Section II	After	15.33	4.66	8.00	25.00	11.00	15.00	18.50	
PARENTS Score	Before	14.38	3.43	6.00	20.00	13.00	14.00	16.50	0.331
Section III	After	13.52	4.43	6.00	21.00	9.00	14.00	17.50	
PARENTS Score	Before	13.24	6.36	6.00	24.00	7.00	10.00	19.00	0.483
Section IV	After	12.38	5.45	4.00	22.00	8.00	11.00	17.50	

Paired T-Test

**Caption:** SD = Standard Deviation; 1Q = First Quartile; 3Q = Third Quartile

In the evaluation carried out by speech language pathologists, there was a significant difference between GI and GII before the intervention in body posture and foot position. After the intervention, there was no difference between them in both characteristics. There was a significant difference between GI and GII after intervention in eye contact and speech velocity (Table 5).

**Table 5:** Association between the assessment of oral presentation and the intervention group in young people

			Group		p-value
			GI	GII	
Body posture (before)	Tense	n	18	8	0.001*
		%	90.0%	38.1%	
	Relaxed	n	2	13	
		%	10.0%	61.9%	
Feet position (before)	Tense	n	16	8	0.011*
		%	80.0%	38.1%	
	Relaxed	n	4	13	
		%	20.0%	61.9%	
Diction (before)	Precise	n	11	17	0.100
		%	55.0%	81.0%	
	Imprecise	n	9	4	
		%	45.0%	19.%	
Voice (before)	Pleasant	n	12	16	0.527
		%	60.0%	76.2%	
	Tense	n	6	4	
		%	30.0%	19.0%	
	Shaky	n	2	1	
		%	10.0%	4.8%	
Intonation (before)	Monotone	n	7	2	0.067
		%	35.0%	9.5%	
	Pleasant	n	13	19	
		%	65.0%	90.5%	
Speech velocity (before)	Adequate	n	17	15	0.454
		%	85.0%	71.4%	
	Fast	n	3	6	
		%	15.0%	28.6%	
Pauses (before)	Adequate	n	7	9	0.751
		%	35.0%	42.9%	
	Filled	n	13	12	
		%	65.0%	57.1%	
Head movement (before)	Adequate	n	6	12	0.162
		%	30.0%	57.1%	
	Tense	n	10	5	
		%	50.0%	23.0%	
	Tilted	n	4	4	
		%	20.0%	19.0%	
Eye contact (before)	Toward the camera	n	16	15	0.719
		%	80.0%	71.4%	
	Down	n	4	6	
		%	20.0%	28.6%	
Body posture (after)	Tense	n	17	18	1.000
		%	85.0%	85.7%	
	Relaxed	n	3	3	
		%	15.0%	14.3%	
Feet position (after)	Tense	n	13	16	0.505
		%	65.0%	76.2%	
	Relaxed	n	7	5	
		%	35.0%	23.8%	
Diction (after)	Precise	n	14	11	0.341
		%	70.0%	52.4%	
	Imprecise	n	6	10	
		%	30.0%	47.6%	
Voice (after)	Pleasant	n	15	15	0.502
		%	75.0%	71.4%	
	Tense	n	4	6	
		%	20.0%	28.6%	
	Shaky	n	1	0	
		%	5.0%	0.0%	
Intonation (after)	Monotone	n	9	9	1.000
		%	45.0%	42.9%	
	Pleasant	n	11	12	
		%	55.0%	57.1%	
Speech velocity (after)	Adequate	n	18	12	0.032*
		%	90.0%	57.1%	
	Fast	n	2	9	
		%	10.0%	42.9%	
Pauses (after)	Adequate	n	9	6	0.341
		%	45.0%	28.6%	
	Filled	n	11	15	
		%	55.0%	71.4%	
Head movement (after)	Adequate	n	10.	7	0.396
		%	50.0%	33.3%	
	Tense	n	8	9	
		%	40.0%	42.9%	
	Tilted	n	2	5	
		%	10.0%	23.8%	
Eye contact (after)	Toward the camera	n	19	13	0.020*
		%	95.0%	61.%	
	Down	n	1	8	
		%	50%	38.1%	

Fisher’s Exact Test and Pearson’s Chi-Squared Test

* Statistically Significant

**Caption:** n = Relative Frequency; % = Percentage Frequency

## DISCUSSION

This study shows that the communication skills training promoted by the method used positively impacted aspects of communication skills of young people and potentially raised the quality of interpersonal communication.

After the training, a difference between GI and GII in the ability to express themselves clearly was observed, which was observed both in the parental assessment and in the self-assessment of the young people. This difference was not verified before the training and can be a direct result of the communication training. Communication skills refer to behaviors that can help individuals better express their feelings and needs and, therefore, achieve their interpersonal goals. By expressing themselves clearly, young people can improve their personal and school relationships, helping with social integration and improving self-confidence. Studies^(21)^ indicate that authentic interpersonal relationships promote the ability to understand one’s own feelings and those of others. In health and work education, such interactions must not be casual, meaning they must have educational objectives to be achieved, since competencies are not established, but built into daily relationships^(21)^.

A similar situation also occurred with the ability to give and receive feedback. After the training, a difference between GI and G II, which was not present before the training, could be observed. This ability can contribute to the development of soft skills, making young people more prepared to face the labor market needs^(3)^. The results of this study based on the responses to the self-assessment and parental assessment questionnaires show that young people who took the training were more adept at giving and receiving feedback and expressing themselves clearly because the training provided them with this self-knowledge through specific strategies that were practiced during the training. Some studies^(15)^ claim that, for example, when a teenager is unable to communicate effectively, frustration or changes in behavior can occur. Any parent of a teenager knows how these skills are part of the parent-teenager relationship. Modeling appropriate communication skills is a great way to show teenagers how people use kind communication to get “what they want”^(15)^.

Several authors^(1,2,9-12,14,15)^ studied the communication skills of doctors and nurses. Communication skills training is part of the curriculum of American medical universities. Interpersonal communication skills form an integrated competency with two distinct parts: communication skills are the performance of specific tasks and behaviors, such as obtaining a medical history, explaining a diagnosis and prognosis, giving therapeutic instructions and advice; and interpersonal skills that are inherently relational and process-oriented and that are the effect of communication on another person, such as relieving anxiety or establishing a trusting relationship.

It is expected that after the training, the communication and interpersonal skills that are trained and learned will help promote this self-knowledge for young people. This will possibly facilitate their professional choice since interpersonal communication is part of social, personal, and professional relationships.

According to the evaluators, there was a difference between groups GI and GII before the intervention in the characteristics of tense body posture and tense feet in the analysis of videos. This difference ceased to exist after the intervention, and an increase in the number of participants with tense body posture and feet position in GII was observed. In addition, after the training, significant differences were observed between G I and G II in speech velocity and eye contact toward the camera, suggesting that G I had significantly changed their corporal conscience during speech. Something as basic as eye contact can be difficult to maintain for many young people and teenagers, although it is the most critical part of nonverbal communication. Looking people in the eye is a skill. It takes practice to understand the importance of eye contact to develop good manners and social connection^(22)^. The improvement in eye contact and speech velocity showed that the training was sufficient to achieve self-confidence and security to express themselves publicly and on camera.

Therefore, to improve the quality of communication in young people, it is recommended that communication skills training is established and taught as a separate course in high school by the related educational authorities. The results of this study can help provide the context and basis for clinical practice in this area.

The present study had some limitations as it was carried out only with students from a private, middle-class school, which can restrict the generalization of the results. Therefore, it is suggested that future research on communication skills’ training, or any other communication method, be carried out with young people who have other sociodemographic characteristics. With the advent of the COVID-19 pandemic, face-to-face training has become more difficult; however, the method can be applied in a virtual environment, thus allowing one more issue to be researched.

## CONCLUSION

The effect of the training on communication skills in young people was able to improve their ability in the clarity of expression and speech, as well as the ability to give and receive feedback, which are essential for the acquisition of other soft skills such as negotiation, teamwork, self-knowledge, and problem-solving. As for the aspects of expressiveness in communication, eye contact and speech velocity were also improved by the method: The participants became aware of the importance of body expressiveness.

Funding Sources: This research did not receive any specific grant from funding agencies in the public, commercial, or not-for-profit sectors.
